# Direct Conversion of Human Fibroblasts into Schwann Cells that Facilitate Regeneration of Injured Peripheral Nerve In Vivo

**DOI:** 10.1002/sctm.16-0122

**Published:** 2017-01-09

**Authors:** Yoshihiro Sowa, Tsunao Kishida, Koichi Tomita, Kenta Yamamoto, Toshiaki Numajiri, Osam Mazda

**Affiliations:** ^1^ Department of ImmunologyGraduate School of Medical Sciences, Kyoto Prefectural University of MedicineKawaramachi Hirokoji Kajii‐cho 465Kamigyo‐kuKyoto602‐8566Japan; ^2^ Department of Plastic and Reconstructive SurgeryGraduate School of Medical Sciences, Kyoto Prefectural University of MedicineKawaramachi Hirokoji Kajii‐cho 465Kamigyo‐kuKyoto602‐8566Japan; ^3^ Department of Plastic and Reconstructive Surgery, Graduate School of MedicineOsaka University 2‐2 Yamadaoka SuitaOsaka565‐0871Japan; ^4^ Department of Dental Medicine, Graduate School of Medical SciencesKyoto Prefectural University of MedicineKawaramachi Hirokoji Kajii‐cho 465Kamigyo‐kuKyoto602‐8566Japan

**Keywords:** Cell transplantation, Glia, Shwann cell, Reprogramming, Transplantation

## Abstract

Schwann cells (SCs) play pivotal roles in the maintenance and regeneration of the peripheral nervous system. Although transplantation of SCs enhances repair of experimentally damaged peripheral and central nerve tissues, it is difficult to prepare a sufficient number of functional SCs for transplantation therapy without causing adverse events for the donor. Here, we generated functional SCs by somatic cell reprogramming procedures and demonstrated their capability to promote peripheral nerve regeneration. Normal human fibroblasts were phenotypically converted into SCs by transducing *SOX10* and *Krox20* genes followed by culturing for 10 days resulting in approximately 43% directly converted Schwann cells (dSCs). The dSCs expressed SC‐specific proteins, secreted neurotrophic factors, and induced neuronal cells to extend neurites. The dSCs also displayed myelin‐forming capability both in vitro and in vivo. Moreover, transplantation of the dSCs into the transected sciatic nerve in mice resulted in significantly accelerated regeneration of the nerve and in improved motor function at a level comparable to that with transplantation of the SCs obtained from a peripheral nerve. The dSCs induced by our procedure may be applicable for novel regeneration therapy for not only peripheral nerve injury but also for central nerve damage and for neurodegenerative disorders related to SC dysfunction. Stem Cells Translational Medicine *2017;6:1207–1216*

**Significance Statement**

Schwann cells (SCs) fulfill important functions in supporting and healing peripheral nerve tissue. Here, we found that transduction of both *SOX10* and *Krox20* genes directly converted human fibroblasts into functional SCs. The directly converted Schwann cells (dSCs) showed typical SC characteristics, and were capable of forming myelin that is the key component of the myelin sheath. Xenogeneic transplantation of the dSCs aided recovery from peripheral nerve injury in mice, leading to functional improvements including locomotive performance. The present technology provides a potential novel transplantation therapy for damaged peripheral and central nervous tissues.

## Introduction

Schwann cells (SCs), the major glial cells in the peripheral nerve system (PNS), have vital roles in the maintenance and regulation of the PNS by secreting neurotrophic factors, producing neuronal extracellular matrix, and accelerating axonal conduction. Immature SCs originate from neural crest cells and differentiate into two distinct mature SC populations, myelinating and the nonmyelinating SCs, that envelope large‐diameter and small‐diameter axons, respectively 1. Krox‐20, Oct‐6, and Sox‐10 are essential transcription factors involved in SC differentiation 2, 3, 4.

SCs also play pivotal roles in neurodegenerative and regenerative processes associated with peripheral nerve injury 5, 6. Transplantation of cultured SCs into an injured nerve site enhanced axonal regeneration across the nerve gap 7, 8. Implantation of a neural prosthesis filled with SCs also facilitated repair of a long segmental gap in the PNS 9. Although the environment in the central nervous system (CNS) is not favorable for the regrowth of nerve fibers, postnatal SC transplantation promoted axonal regeneration of lesioned adult rat spinal cord 10, 11, 12. Transplantation of SCs derived from adult human nerve remyelinated the demyelinated axons in the CNS and restored the conduction properties of the damaged nerve 13. Therefore, transplantation of SCs may provide a considerable therapeutic benefit to patients with PNS and CNS injuries, including a large nerve defect caused by trauma and by surgical resection of a tumor such as a sarcoma or an advanced dermal tumor.

A major problem is the difficulty in obtaining enough number of functional SCs for transplantation. To prepare autologous or allogenic SCs for such transplantation therapy, a normal nerve (such as the sural nerve and great auricular nerve) has to be resected from the patient or from an allogenic donor as a source of the SCs. Since cultured SCs have a restricted growth potential, a certain amount of the nerve tissue is required as the starting material to provide a sufficient number of SCs for the transplantation. Such sacrifice of a nerve may cause some adverse events, including pain and paralysis, to the patient or donor. To overcome this problem, we have tried to establish a novel technology to generate a large number of functional SCs from somatic cells that can be obtained from either a patient or a donor without an invasive procedure.

Recent studies in the field of cellular reprogramming have enabled conversion of somatic cells into specific differentiated lineages without passing through an intermediate pluripotent state, by transducing a particular set of transcription factor genes. The resultant cell lineages include cardiomyocytes 14, 15, neurons 16, 17, chondrocytes 18, 19, hematopoietic cells 20, myocytes 21, Sertoli‐like cells 22, and hepatocytes 23. We previously reported direct conversion of human fibroblasts into osteoblasts 24 and brown adipocytes 25. These procedures may allow production of various tissue cells that are not tumorigenic and are suitable for transplantation therapies for a variety of diseases and injuries.

In the current study, we aimed to directly convert human fibroblasts into SCs by introducing genes encoding SC‐specific transcription factors. We also assessed myelin formation and repair of peripheral nerve injury in vivo by the directly converted Schwann cells (dSCs).

## Materials and Methods

### Cells

Normal human dermal fibroblasts (aHDFs) and a PLAT‐GP packaging cell line were purchased from Cell Biolabs (cat no. KF‐4109) and from Kurabo (cat no. VPK‐305), respectively. The cells were cultured in Dulbecco's modified Eagle's medium (DMEM) supplemented with 10% fetal bovine serum (FBS), 100 mM nonessential amino acids, 100 U/ml penicillin, and 100 μg/ml streptomycin (Complete medium). Human primary Schwann cells (pSCs) were obtained as described previously 26. Briefly, nerve tissue was excised from three patients who underwent breast reconstructive surgery after giving informed consent. After removing the epineurium, the nerve was cut into 1‐mm segments that were subsequently cultured in DMEM supplemented with 5 ng/ml platelet‐derived growth factor (PeproTech Ltd., London, UK), 10 ng/ml basic fibroblast growth factor (b‐FGF) (PeproTech), 5.7 ng/ml forskolin (Sigma Aldrich, St. Louis, MO, USA), and 200 ng/ml recombinant human heregulin‐b1 (HRG‐b1) (R&D Systems, Minneapolis, MN, USA) (SC medium). Two weeks later, the medium was removed, and 0.0625% collagenase type IV and 0.585 U dispase were added to the dish. After incubation for 24 hours, the cell suspension was filtrated through a 70‐μm cell strainer, followed by centrifugation. The cell pellet was resuspended in SC medium and plated onto a 100‐mm dish that had been coated with laminin (Sigma Aldrich). The culture was maintained until the cells reached subconfluency.

### Antibodies

The following antibodies were used as primary antibodies for immunocytostaining and for immunohistochemistry: rabbit anti‐s100b (dilution = 1:1,000) (Dako, Ely, UK), rabbit anti‐p75 (1:500) (Cell Signaling Technology, Danvers, MA, USA), rabbit anti‐GAP43 (1:200) (Cell Signaling Technology), rabbit anti‐glial fibrillary acidic protein (GFAP) (1:200) (Abcam, Cambridge, UK), rabbit anti‐NG2 (1:1,000) (Millipore, Cambridge, UK), mouse anti‐myelin basic protein (MBP) (1:100) (Chemicon, Chandlers Ford, UK), goat anti‐protein zero (P0) (1:500) (Abcam), rabbit anti‐Tuj‐1 (1:100) (Chemicon), mouse anti‐neurofilament (NF) (1:1,000) (Sigma), rabbit anti‐PGP9.5 (1:500) (Abcam), mouse anti‐fibronectin (Millipore), mouse anti‐Nanog (1:200) (ReproCELL, Yokohama, Japan), mouse anti‐MAP‐2 (1:200) (Abcam), and rabbit anti‐NeuN (1:500) (Abcam) antibodies. As secondary antibodies, Alexa Fluor 546‐conjugated anti‐rabbit and anti‐mouse antibodies and Alexa Fluor 670‐conjugated anti‐rabbit and anti‐mouse antibodies (1:1,000) (Life technologies, Carlsbad, CA, USA) were used.

### Retroviral Vectors

The coding sequences for human *SOX10*, *Krox20*, and *Oct6* genes (DNAFORM cDNA clone library; DNAFORM) were cloned into the EcoRI‐digested pMXs vector (Cell Biolabs). The pMXs vectors containing human *Kruppel‐like factor 4 (KLF4)*, *Oct4*, *c‐Myc*, and *GFP* genes were generously provided by Professor S. Yamanaka (Kyoto University) 27. Retroviral vectors were freshly prepared for each experiment as described elsewhere 28. Briefly, PLAT‐GP packaging cells (5.5 × 10^6^) were seeded onto gelatin‐coated 100‐mm dishes and cultured overnight. pCMV. VSV‐G and one of the above‐mentioned pMX plasmids containing the transcriptional factor genes were diluted in Opti‐MEM and cotransfected into the cells using the X‐treme Gene 9 transfection reagent (Roche Applied Science). Twenty‐four hours later, the culture medium was replaced with anti‐biotic free culture medium. After culturing for another 24 hours, the medium was collected and filtrated through a 0.45‐mm pore‐size filter. Every virus suspensions contained comparably high titers of the retrovirus vectors (5.6–6.0 × 10^6^/ml) as demonstrated by using the QuickTiter Retrovirus Quantitation Kit (Cell Biolabs) (Supporting Information Fig. S1).

### Infection and SC Induction

HDFs were seeded onto culture dishes or plates at a density of 2.0 × 10^4^ cells per ml. After culturing overnight, the cells were transduced with various combinations of retroviral vectors in the presence of 4 µg/ml polybrene (day 0). On day 1, the culture medium was replaced with SC medium. The culture medium was replaced every 2 days. All transgenes were expressed at comparably high levels in the cells transduced with a mixture of the retroviral vectors (Supporting Information Figs. S2 and S3). After transduction with the *GFP* retroviral vector, more than 95% of the cells strongly expressed GFP (Supporting Information Fig. S4).

### Immunocytochemistry

Cells were fixed in 4% paraformaldehyde (PFA) for 30 minutes, followed by permeabilization using 1% Triton‐X 100 at room temperature for 20 minutes. With regard to immunostaining of MBP and P0 antigens, the cells were incubated in ice‐cold methanol for 10 minutes before permeabilization. For Nanog immunostaining, the cells fixed in paraformaldehyde were subsequently permeabilized with 0.2% Triton X‐100 for 15 minutes at room temperature. Background staining was blocked by incubation with normal goat serum (Nakalai Tesque, Kyoto, Japan) for 1 hour at room temperature, followed by incubation of the cells with primary antibodies at 4°C overnight. Cells were washed and incubated with secondary antibodies for another 1 hour at room temperature. After washing three times, cells were mounted and observed under a fluorescence microscope (IX71; Olympus, Tokyo, Japan). More than 100 cells were differentially counted based on the SC‐like morphology and expression of glial markers.

### Morphological Analysis

The aspect ratio (length vs. width) of cells was assessed as previously described 26, 29. Briefly, HDFs and pSCs were seeded into 8‐well Lab‐tek chamber slides (Thermo Scientific, Waltham, MA, USA) at a low density (2 × 10^4^/ml). The HDFs in some chambers were converted into dSCs by retroviral transduction of SK genes and subsequent culturing in SC medium for 10 days. HDFs were immunostained with anti‐fibronectin antibodies, while pSCs and dSCs were stained with polyclonal anti‐s100b antibody as above. The slides were observed under a fluorescence microscope, and more than 15 cells were examined for each group. The mean aspect ratios from three independent experiments were calculated.

### Real‐Time Reverse Transcription Polymerase Chain Reaction (RT‐PCR)

RNA was extracted using ISOGEN II (Nippon Gene, Tokyo, Japan) and reverse transcribed using ReverTra Ace qPCR RT Master Mix (TOYOBO Life Science, Tokyo, Japan). Quantitative PCR was carried out using Real‐time PCR Master Mix (TOYOBO) and the matching probes/primers shown in the Supporting Information Table 1 on a 7300 Real‐Time PCR System (Applied Biosystems, Foster City, CA, USA). The relative mRNA levels (average ± SD) were calculated as follows: Relative mRNA level (fold) = ([target gene mRNA level in sample]/[β‐actin gene mRNA level in sample])/([target gene mRNA level in control]/[β‐actin gene mRNA level in control]).

### ELISA

Cells were seeded in 12‐well plates at 2 × 10^5^/1,000 μl complete medium/well (*n* = 3). After culturing for 48 hours, the culture media were harvested, and the concentrations of brain‐derived neurotrophic factor (BDNF), nerve growth factor (NGF), and glial cell‐derived neurotrophic factor (GDNF) were measured using the Emax ImmunoAssay System (Promega, Madison, WI, USA).

### Coculture of SCs and NG108‐15 Neuronal Cells

HDFs, pSCs, and dSCs were seeded into the chamber slides at 5 × 10^3^ cells per 100 mm^2^ chamber and cultured for 24 hours. NG108‐15 neuronal cells were added at 3 × 10^3^ per chamber and cocultured in DMEM/Ham's F12 plus 2% B27 supplement (GIBCO‐BRL, Grand Island, NY, USA) for another 24 hours. The cells were fixed and incubated with anti‐MAP‐2 and anti‐NeuN antibodies, followed by staining with Alexa Fluor 546‐conjugated anti‐mouse and Alexa Fluor 488‐conjugated anti‐rabbit antibodies and DAPI as described above. The slides were examined using an Olympus BX71 fluorescence microscope, and neurite outgrowth analysis was performed using the Image J digital image analysis program. The percentage of neuron‐bearing neurites, the longest neurite length, and the number of neurites per neuron were calculated. A total of 20 neuronal cells were examined for each sample, and the means of three independent experiments are reported.

### Coculture of SCs and Dorsal Root Ganglion

To prepare *GFP*‐labeled dSCs, aHDFs were infected with a mixture of *SOX10*, *Krox20*, and *GFP* retroviral vectors and cultured in SC medium for 4 days. *GFP*‐labeled pSCs were produced by infecting pSCs with *GFP* retroviral vector and cultured for 4 days. Dorsal root ganglions (DRGs) were obtained from mice and dissociated as previously described 30. Briefly, 2,000 neurons were plated on 0.02% laminin (Sigma)‐coated chamber slides and cultured in Eagle's minimum essential medium supplemented with 4 g/l D‐glucose, 50 ng/ml NGF, 10% horse serum, 2 mM L‐glutamine, 100 U/ml penicillin, and 100 μg/ml streptomycin (DRG growth medium). Twenty‐four hour later, proliferating non‐neuronal cells were eliminated from the DRG cell population by culturing them in DRG Purification Medium (EMEM supplemented with 4 g/l D‐glucose, 10 μM Arabinocytidine (Ara‐C), 10 μM deoxyuridine, 50 ng/ml NGF, 1 × N2 supplement, 2 mM L‐glutamine,100 U/ml penicillin, and 100 μg/ml streptomycin) for 3 days. The *GFP*‐labeled pSCs and dSCs were then added to the DRG culture at a density of 2 × 10^5^ cells per chamber and further cultured in DMEM supplemented with 4 g/l D‐glucose, 50 ng/ml NGF, 50 μg/ml ascorbic acid, 5% HS, 2 mM L‐glutamine, 1 × N2 supplement, 20 μg/ml bovine pituitary extract, 0.5 μM forskolin, 100 U/ml penicillin, and 100 μg/ml streptomycin as previously described 31. Two days later, 50 μg/ml ascorbic acid was added, and the coculture was continued for an additional 2 weeks before immunocytochemical observation.

### Surgical Procedure and Cell Transplantation

Every animal experiment was approved by the institutional Animal Experiment Committee, and the care of the animals was in accordance with our institutional guidelines. Creation of the sciatic nerve injury and cell transplantation were performed as described with a slight modification 32. Briefly, pSCs and dSCs were allowed to adhere onto the inner surface of a gelatin hydrogel conduit at 10^4^ cells per tube. Eight‐week‐old NOD/SCID (CLEA Japan, Tokyo) and Balb/c nu/nu mice weighing about 25 g were anesthetized, and a longitudinal incision was made at the lower limb in parallel with both the extension of the Achilles tendon and gastrocnemius muscle. A segmental sciatic nerve defect was created (the length of the defect was 5 mm), and the proximal and distal termini were inserted into the gelatin hydrogel conduit containing either pSCs or dSCs (*n* = 4 each) in such a manner that the termini were 5 mm apart from each other. In some experiments, pSCs and dSCs infected with GFP retrovirus vector before transplantation were used. The control group (*n* = 4) received a nerve guide conduit filled with SC culture medium. Sham operated mice received the same surgical procedure except for the lack of creation of the nerve defect and transplantation.

### Immunohistochemistry

Twelve weeks after the surgery, the mice were anesthetized, and the gastrocnemius muscle and Achilles tendon were excised from both the experimental and control limbs. The operated sciatic nerves were fixed in 4% PFA, cryoprotected in 20% sucrose PBS, and freeze‐mounted. Longitudinal cryosections were cut into 5‐µm thick sections, and immunostaining was performed as described above.

### Estimation of Myelinated Axons

The specimens were cross‐sectioned at the proximal third of the graft, fixed in 10% buffered formalin solution, and embedded in paraffin. Five‐micrometer sections were stained with Luxol blue dye. Fluorescence and optical light microscopic images were captured by BZ‐9000 (Keyence) with ImageJ software. A total of five images were analyzed per each cross‐section, and the density of the myelinated fibers was determined as the number of myelinated nerve fibers per 1.0 mm^2^. The total number of myelinated axons was calculated using ImageJ software.

### Estimation of Muscle Mass

The gastrocnemius muscle was excised from both the affected and unaffected limbs of the mice and weighed. The muscle mass ratio for each animal was calculated as follows. Muscle mass ratio = % (muscle mass of the affected side)/(muscle mass of the unaffected side) 33.

### Walking Track Analysis

Six and 12 weeks after the surgery, all animals were subjected to a walking‐track analysis as described 34. The sciatic functional index (SFI) was calculated as follows: SFI = 118.9 × ([ETS‐NTS]/NTS) − 51.2 × ([EPL‐NPL]/NPL) – 7.5, where ETS, NTS, EPL, and NPL represent experimental toe spread, normal toe spread, experimental print length, and normal print length, respectively.

### Statistical Analysis

All data are expressed as means ± SD. One‐way ANOVA and Tukey's multiple comparison tests were used to compare differences between groups. All analyses were conducted with InStat 3.0 software (GraphPad, La Jolla, CA, USA). Differences were considered significant at *p* < .05.

## Results

### Cotransduction of SOX10 and Krox20 Genes Potently Induces SC Phenotypes in Human Fibroblasts

We initially focused on three transcription factors, *SOX10*, *Oct6*, and *Krox20*, that are crucial regulators of SC differentiation, as well as four reprogramming factors, *Oct4*, *KLF‐4*, *SOX2*, and *c‐Myc* due to their potential for modifying cell fates 27, 35. Retroviral vectors encoding these factors were infected into human fibroblasts either individually or in various combinations. The SC‐like phenotype was assessed by expression of the s100b protein that is broadly expressed in almost all of the differentiation stages of SCs 36, 37, 38, 39, 40. As shown in Supporting Information Figure S5, the s100b‐positive cells were strongly induced after cotransduction of the two genes, *SOX10* plus *Krox20* (No. 4). Some combinations that contained both *SOX10* plus *Krox20* genes (No. 7, 53–67, and 98–112) also induced the SC marker in the fibroblasts. Other combinations that contained *SOX10* and *Oct6* but not *Krox20* (No. 5 and 68–82) induced s100b expression at low levels.

**Figure 1:**
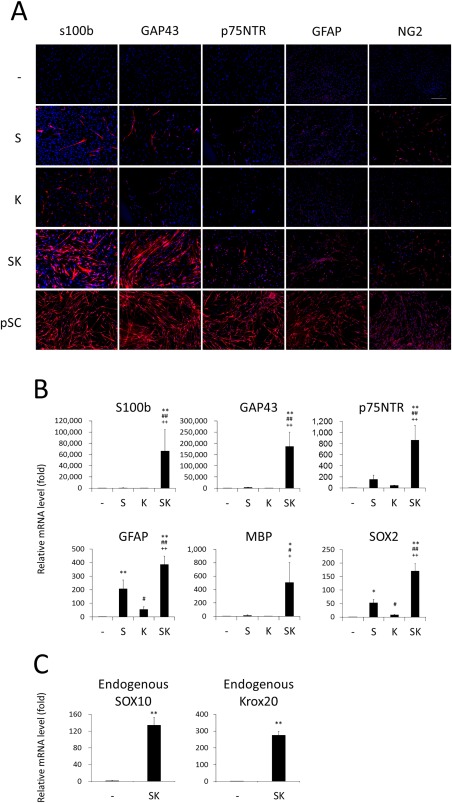
Schwann cell (SC)‐like phenotypes were induced in human dermal fibroblasts by transduction of two transcription factor genes normal human dermal fibroblasts were transduced with the *S* and/or *K* retroviral vectors and cultured in SC medium. **(A)**: Ten days later cells were immunostained with the indicated antibodies. Human primary Schwann cells (pSCs) were also stained as a control. Representative fluorescence microscopic images are shown. *n* = 3 cultures per group. Three independent experiments were performed. Scale bar = 500 µm. (**B, C**): mRNA levels for the indicated genes were evaluated by real‐time RT‐PCR, 10 (B) and 14 (C) days after the transduction. Values are means ± SD. *n* = 6 cultures. *p < .01 and **p < .001 versus Control; #*p* < .01 and ##*p* < .001 versus S; and +*p* < .01 and ++*p* < .001 versus K. Abbreviations: GFAP, glial fibrillary acidic protein; MBP, myelin basic protein.

Thus, we transduced *SOX10* (*S*) and/or *Krox20* (*K*) genes into fibroblasts and examined the cells in more detail. After culturing in SC medium for 10 days, a small proportion (approximately 4%) of the cells that had been transduced with *SOX10* alone expressed s100b, while another typical SC marker, GAP43, was also induced in the *SOX10*‐transduced cells (Fig. 1A). In contrast, transduction of both *SOX10* and *Krox20* (*SK*) induced a much larger proportion of the cells that expressed high levels of these SC‐related proteins (s100b‐ and GAP43‐positive cells accounted for 43.5% ± 5.3% and 37.9% ± 7.5% of the *SK*‐transduced cells, respectively). A considerable proportion of the *SK*‐transduced cells were also positive for other SC markers, including p75NTR, GFAP, and NG2 [38].

The cells transduced with *SOX10* and/or *Krox20* were tested for the expression levels of mRNA for the SC‐related genes. Quantitative RT‐PCR analysis revealed that the transduction of SOX10 alone induced mRNA for *p75NTR*, *GFAP*, and *SOX2* at low to intermediate levels, whereas the SK‐transduced cells highly expressed *s100b*, *GAP43*, *p75NTR*, *GFAP*, and *SOX2* mRNA (Fig. 1B). Moreover, *SK* transduction also induced mRNA expression of the myelin‐related gene, *MBP*. The *SK*‐transduced cells significantly expressed mRNA for endogenous *SOX10* and *Krox20* genes (Fig. 1C). Based on these findings, we defined the *SK*‐transduced cells as dSCs and characterized them in more detail in additional experiments.

**Figure 2:**
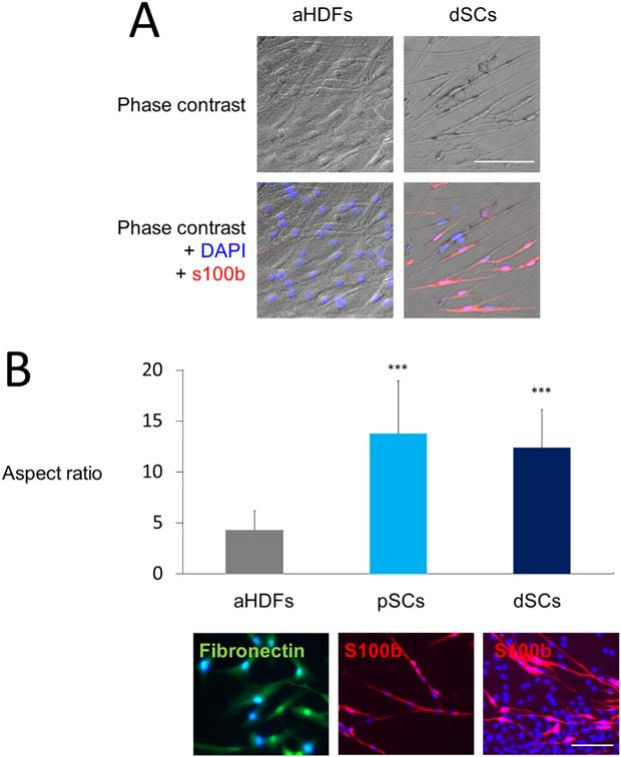
Morphological and phenotypic characteristics of the directly converted Schwann cells (dSCs) **(A)** normal human dermal fibroblasts (aHDFs) were transduced with *S* and *K* retroviral vectors and cultured for 2 weeks. dSCs and nontransduced aHDFs were stained with both DAPI and anti‐s100b antibody. Representative phase contrast (upper) and fluorescent (lower) images are shown. Scale bar = 100 µm. **(B)**: Nontransduced aHDFs were seeded at a density of 2 × 10^4^/ml and stained with an anti‐fibronectin antibody, while pSCs and dSCs cultured at the same density were stained with an anti‐s100b antibody, to visualize cell shape. Representative fluorescence microscopic images (lower; Scale bar = 50 µm) and the aspect ratios (means ± SD, *n* = 3 experiments) (upper) are shown. ***p < .001 versus aHDFs. Abbreviations: dSCs, directly converted Schwann cells; aHDFs, normal human dermal fibroblasts.

The dSCs showed typical spindle‐shaped morphology (Fig. 2A). SCs exhibit high aspect ratios (cell length vs. cell width), which has been considered a typical feature of the SCs 26, 29. Thus we estimated the aspect ratios of aHDFs, dSCs, and pSCs. The aspect ratio of aHDFs was 4.3 ± 1.9, whereas those of pSCs and dSCs were 13.8 ± 5.1 and 12.4 ± 3.7, respectively (Fig. 2B). The data strongly suggest that SK transduction and subsequent culturing in SC medium resulted in significant elongation of the cell shape (Fig. 2B).

dSCs strongly expressed retroviral *SOX10* and *Krox20* genes (Supporting Information Fig. S6). To elucidate whether continuous expression of the transgenes are required for the maintenance of the SC phenotypes, we transfected aHDFs with a plasmid vector encoding *SOX10* and *Krox20* genes and cultured them in the SC medium. As results, dSCs were successfully induced (Supporting Information Fig. S7A), while the plasmid vectors had been lost from the cells (Supporting Information Fig. S7B). The findings strongly suggest that transient expression of exogenous *SOX10* and *Krox20* genes are sufficient for the conversion of fibroblasts into dSCs.

We also examined whether SC medium is essential for the cell conversion. Fibroblasts were infected with *SOX10* and *Krox20* retroviral vectors and cultured in DMEM. The cells were successfully converted into dSCs, albeit less efficiently than the *SK*‐transduced cells cultured in the SC medium (Supporting Information Fig. S8).

Other than fibroblasts, human adipose stromal cells and umbilical vein endothelial cells were also converted into dSCs by retrovirus vector‐mediated transduction of *SK* (Supporting Information Fig. S9).

### dSCs Secrete Neurotrophic Factors and Promote Neurite Outgrowth at Comparable Rates to Those of pSCs

**Figure 3:**
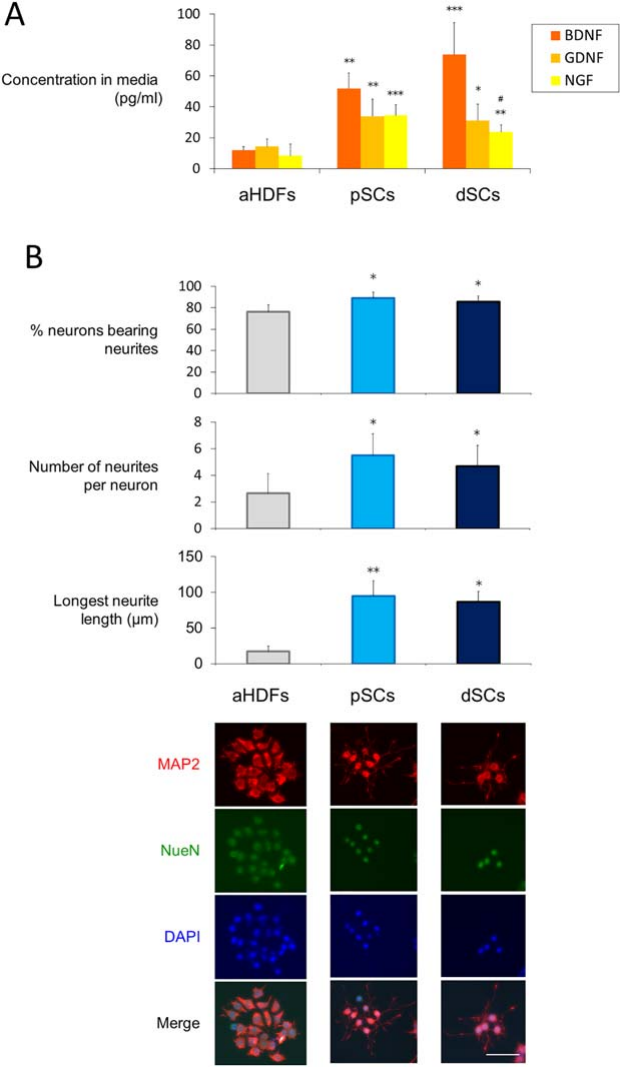
Directly converted Schwann cells (dSCs) significantly produced neurotrophic factors and induced neurons to produce neurites **(A)** Cells (2 × 10^5^ per well) were cultured for 48 hours, and the conditioned medium was analyzed by ELISA to determine the concentrations of the indicated neurotrophic factors. The data are expressed as means ± SD (*n* = 6 experiments). **p < .01, ***p < .001 versus normal human dermal fibroblasts (aHDFs), ^#^ *p* < .05 versus pSCs. **(B)**: The indicated cells were cocultured with NG108‐15 neuronal cells for 24 hours. NG108‐15 cells were immunostained with anti‐MAP2 and anti‐NeuN antibodies, followed by Alexa 488‐ and Alexa Fluor 546‐conjugated secondary antibodies to visualize the neurites. Cell nuclei were stained with DAPI. Representative fluorescent microscopic images are shown (lower) (Scale bar = 50 µm). The percentage of neurite‐bearing neurons, the number of neurites per neurons, and the longest neurite length were calculated using ImageJ software (20 cells were analyzed for each group) (upper). The data are expressed as means ± SD (*n* = 4 cultures), *p < .05, **p < .01 versus aHDF culture. The experiments were repeated three times. Abbreviations: aHDFs, normal human dermal fibroblasts; BDNF, brain‐derived neurotrophic factor; dSCs, directly converted Schwann cells; GDNF, glial cell‐derived neurotrophic factor; NGF, growth factor.

As a functional assessment of dSCs, we examined whether dSCs produced three typical neurotophic factors, that is, BDNF, NGF, and GDNF. ELISA demonstrated significantly higher concentrations of the three factors in the culture medium of dSCs than those in the culture medium of aHDFs (Fig. 3A). Compared with pSCs, the dSCs produced a significantly lower level of GDNF and a higher level of BDNF, while NGF was produced at similar levels by these cells.

Another important function of SCs is the induction of neurite outgrowth of neurons. aHDFs, dSCs, and pSCs were cocultured with NG108‐15 neuronal cells, and the number and length of neurites were evaluated by immunostaining the neuronal cells with anti‐Tuj‐1 antibody. As shown in Figure 3B, the dSCs enhanced neurite outgrowth at a comparable degree to that of pSCs, as revealed by statistical analyses of three criteria, that is, the proportion of neurite‐bearing cells, number of neurites per cell, and length of the longest neurite in each cell. In contrast, the nontransduced aHDFs enhanced neurite outgrowth very faintly, if at all.

### dSCs Produce Myelin In Vitro and In Vivo

**Figure 4:**
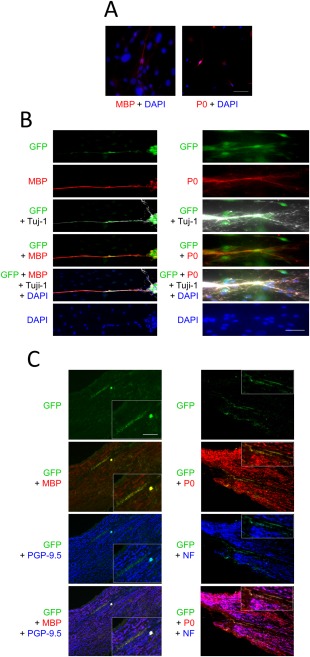
Directly converted Schwann cells (dSCs) formed myelin both in vitro and in vivo **(A)** dSCs were stained with both anti‐myelin basic protein (MBP) and anti‐P0 antibodies and with DAPI. Scale bars = 100 µm. **(B)**: *GFP*‐transduced dSCs were cocultured with primary rat dorsal root ganglion (DRG) for 14 days, followed by staining with an anti‐Tuj‐1 antibody (gray) and with either anti‐MBP (left) or P0 (right) antibody (red). Scale bars = 100 µm. **(C)**: *GFP*‐labeled dSCs were transplanted into the sciatic nerve defect of nude mice. Twelve weeks later the sciatic nerve tissue was excised and longitudinal sections were immunostained with anti‐MBP (red) and anti‐PGP‐9.5 (blue) antibodies (left) or with anti‐P0 (red) and anti‐NF (blue) antibodies (right). The arrowheads indicate the co‐localization of GFP and MBP (left) and of GFP and P0 (right) signals. Scale bar = 500 µm. The inner panels show higher magnification images of the indicated area in the main panels (Scale bar = 20 µm). Abbreviations: GFP, green fluorescence protein; MBP, myelin basic protein; NF, neurofilament.

dSCs in culture expressed myelin‐related proteins, MBP and Protein Zero (P0), at very low levels (Fig. 4A). We hypothesized that dSCs may differentiate into myelinating SCs after interaction with a neuron, and examined this possibility by using the DRG/SC direct‐contact coculture system. The dSCs were labeled with GFP to distinguish them from endogenous SCs that were possibly derived from the DRG. We examined whether dSCs came in contact with neurites and expressed myelin proteins.

During the coculture, some *GFP*‐labeled dSCs gradually migrated to and aligned with the Tuj‐1‐positive DRG neurites. Some of the GFP‐positive dSCs that were in contact with an axon expressed both MBP and P0 that are major membrane proteins of the mature myelin sheath (Fig. 4B). Similar phenomena were also seen in the coculture of the pSCs and DRG. The density of the myelinated fragments was 5.5 ± 0.3/mm^2^ in dSC plus DRG coculture, while that in pSC plus DRG coculture was 9.3 ± 2.1/mm^2^ (Supporting Information Fig. S10). The results strongly suggested that dSCs were actively involved in myelination of the axons, albeit at a lower degree than pSCs.

We investigated whether dSCs interacted with regenerating axons and formed myelin in vivo. A segmental defect was made in the sciatic nerve of nude mice, and *GFP*‐labeled dSCs were transplanted into the lesion. Twelve weeks later, the GFP‐positive dSCs were densely populated at the distal regions of the nerve, where MBP and P0 proteins were also located (Fig. 4C). To clarify the detailed distribution of the transplanted cells, regenerating nerves, and myelin, the axons and myelin protein were immuno‐stained with anti‐PGP‐9.5 and MBP antibodies, respectively. Most GFP‐positive cells aligned with the PGP‐9.5‐positive regenerating axons where MBP exactly colocalized with these axons (Fig. 4C, left). Similar findings were also obtained by staining for NF and for P0 proteins to visualize regenerating nerves and myelin, respectively (Fig. 4C, right). Taken together, dSCs survived at the transplant site, made contact with the regenerating axons, and formed myelin in vivo.

### Transplantation of dSCs Enhances Peripheral Nerve Regeneration and Improves Neural Functions

**Figure 5:**
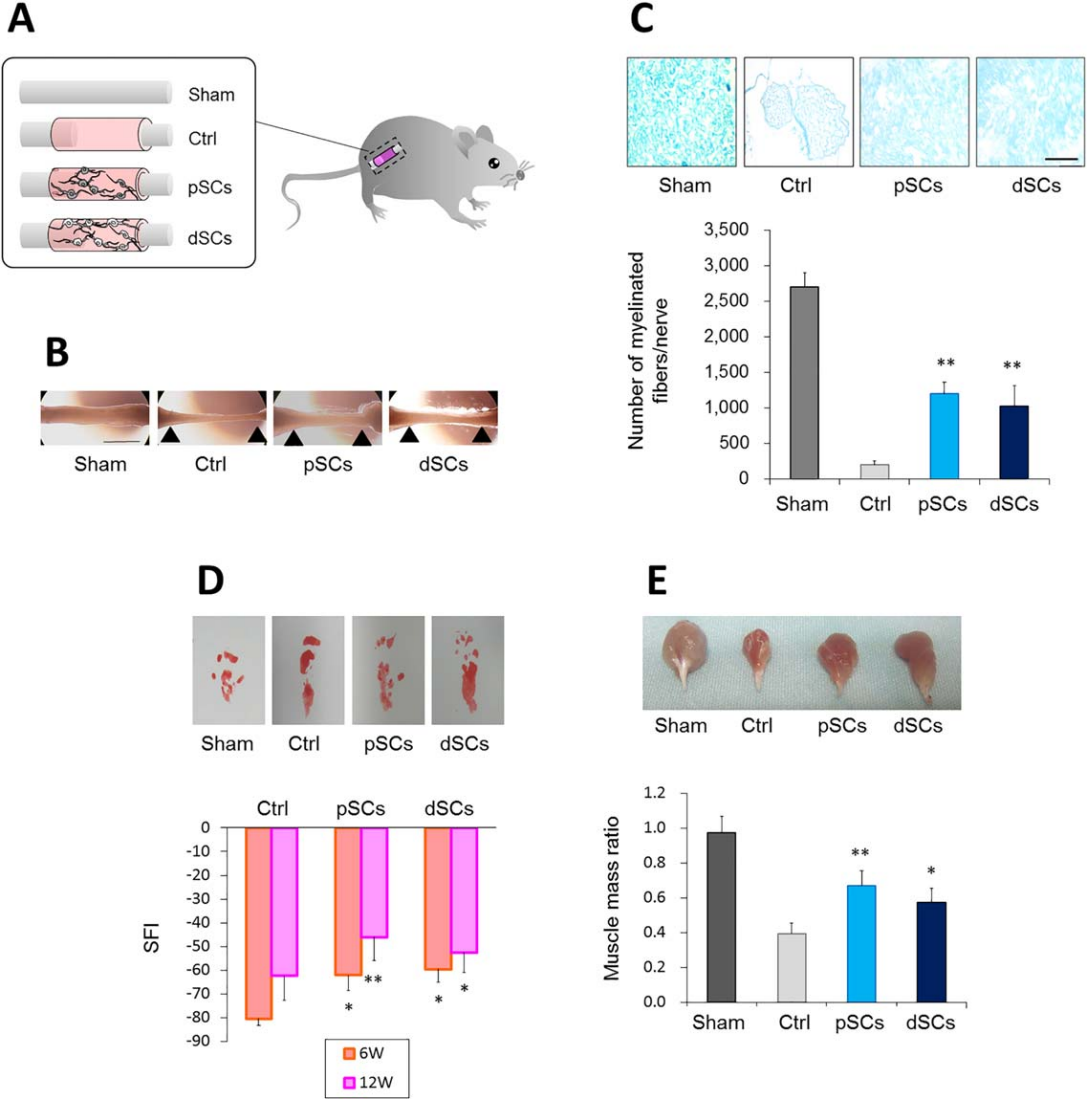
Transplanted directly converted Schwann cells significantly enhanced regeneration of an artificial peripheral nerve defect in mice **(A)** Schematic view of an artificial peripheral nerve defect, tubulization, and cell transplantation. **(B)**: Twelve weeks after surgery, the graft region was excised. Macroscopic views of the sciatic nerves are shown. Arrow‐heads indicate stump anastomosis sites. Scale bar = 5.0 mm. **(C)**: Nerve defect lesions were excised 8 weeks after the surgery, and transverse sections of the tissues were prepared. Myelin was stained with Luxol fast blue (upper). Scale bar = 500 µm. The number of myelinated fibers per nerve is plotted (lower). *n* = 4 mice per group. **p < .001 versus Control. **(D)**: Six and 12 weeks after the surgery, mice were subjected to functional analysis by the walking track method. Representative hind feet tracks of mice recorded 6 weeks after the surgery are shown (upper), while SFI at the indicated periods are calculated (lower). Values are means ± SD. *p < .01 and **p < .001 versus Control. **(E)**: Twelve weeks after the surgery, the gastrocnemius muscle was excised from the affected and unaffected limbs. Representative macroscopic images of the muscle from the affected limbs (upper) and muscle mass ratios (lower) are shown. Values are means ± SD. *p < .01 and **p < .001 versus Control. Abbreviation: dSCs, directly converted Schwann cells.

Finally, the potential of dSCs to regenerate an injured peripheral nerve was examined. We transplanted dSCs and pSCs within nerve guide conduits into segmental nerve defect lesions surgically created at the sciatic nerve of NOD/SCID mice (Fig. 5A). Twelve weeks later, continuity of the nerve was observed, but the regenerated nerve was very thin, in the control group that had been transplanted with a cell‐free gelatin conduit (Fig. 5B). In contrast, the nerve defect was more completely repaired in the pSC and dSC transplanted groups, and the diameter of the regenerated nerve was much larger than that in the control group. We also assessed myelination of the regenerated nerve. As shown in Figure 5C, in the pSC‐ and dSC‐transplanted groups, Luxol fast‐staining myelin was broadly distributed in the regenerated nerve tissue, and the myelinated nerve fibers were significantly higher in numbers compared with that in the control group (approximately 5.9 times and 5.0 times, respectively) (Fig. 5C).

We also analyzed the functional recovery of the injured sciatic nerve. Walking track analysis revealed that pSC and dSC groups showed more significant improvement of the locomotive performance than the cell‐free group (control) at both 6 and 12 weeks postinjury. No significant difference was found between the pSC and dSC groups (Fig. 5D).

Since the posterior tibial branch of the sciatic nerve innervates the gastrocnemius muscle, transection of the sciatic nerve results in atrophy of this muscle, while the gastrocnemius muscle regains its mass depending on the amount of axonal reinnervation 32. The weight of the gastrocnemius muscle indicated that the transplantation of pSCs and dSCs within nerve guide conduits significantly reversed the muscle atrophy (Fig. 5E), suggesting that the dSC transplantation boosted reinnervation to restore muscle atrophy as did the pSCs. These results strongly suggested that dSC transplantation improved motor function of the animals as significantly as that observed with pSC transplantation.

## Discussion

Cell reprogramming technologies have great potential to provide a variety of tissue‐specific cells for transplantation without causing severe adverse events to the donors from whom fibroblasts can be used for autografts or allografts. In this study, we directly converted human fibroblasts into SCs by transduction of two defined transcription factors, *SOX10* and *Krox20*. The resultant dSCs had typical SC‐like morphology and functions, including myelin formation, secretion of neurotrophic factors, and stimulation of neurite outgrowth from neuronal cells. Furthermore, the dSCs that were transplanted into a peripheral nerve injury site expressed myelin protein and facilitated regeneration of axons, indicating their potent glial function in vivo.

Although molecular regulation of SC development has not been fully understood, Sox10, Oct6, and Krox20 are known to play central roles in the induction of SC differentiation 2, 3, 4. Sox10, a SRY‐related high‐mobility group (HMG) domain protein, is a master transcriptional regulator involved in SC development and is highly expressed at all stages of the SC lineage 41. Sox10 resides upstream of the gene network that determines SC fate and controls the target genes that contribute to the maintenance and regulation of the PNS 42, 43. In *Sox10* deficient mice, neural crest cells were not able to differentiate into SC precursors, indicating that this transcription factor is required for SC specification 44. Krox20 is a zinc finger transcription factor. It is expressed at later stages of SC development, and its expression reaches the highest level during the final maturation of SCs from their precursors 45. Topilko et al. suggested that lack of Krox20 caused differentiation arrest of the SC lineage cells at the promyelin stage 46. Interestingly, Oct6 is another key transcription factor essentially involved in the differentiation of SCs 47, 48, 49, but Oct6 was not required for the direct conversion of fibroblasts into SCs. An important role of Oct6 in SC differentiation is its ability to induce Krox20 expression 47, 48, 49. Thus, transduction of exogenous Krox20 may have eliminated the requirement of Oct6 for SC conversion.

With our procedure, approximately 43% of adult fibroblasts were converted into dSCs (Fig. 1A). The conversion efficiency is higher than those in previous studies on direct reprogramming, in which 0.24%–28% of fibroblasts were successfully converted into various cell lineages, such as cardiomyocytes, hepatocytes, neurons, and chondrocytes 14, 15, 16, 17, 18, 19, 20, 21, 22, 23. The SC‐lineage cells at different maturation stages express various SC markers at different levels 50, 51, 52. The dSCs expressed some SC markers (such as s100b and GAP43) at high levels but others (such as GFAP and NG2) at low levels (Fig. 1A). Therefore, dSCs may contain heterogenous SC‐lineage cells at various developmental stages.

Recently, Thoma et al. induced transdifferentiation of human fibroblasts into SCs by culturing the cells with chemical compounds 40. The process involved two steps: initial conversion of fibroblasts into neural precursors and subsequent differentiation of the neural precursor into SCs. Thus, the mechanism of transdifferentiation may be different from that underlying our direct conversion. In contrast to our dSCs, it has not been clarified whether the SCs induced by their method promote regeneration of injured nerve in vivo, which is quite important for assessing physiological activities of the SCs.

Our present findings may translate to clinical applications for not only peripheral nerve injury but also for brain and spinal cord injury and for demyelinated CNS disorders, including multiple sclerosis (MS). SCs form myelin in the CNS 12, 13. In MS, autoimmune reactions cause the death of oligodendrocytes and degradation of the CNS myelin formed by oligodendrocytes, whereas the PNS myelin formed by SCs remains unaffected 49. Transplantation of autologous dSCs may result in remyelination of demyelinated CNS axons in MS patients, as suggested by some previous animal studies in which peripheral nerve‐derived SCs formed myelin in MS lesions 53. Functional aberrations of SCs are also important in both the pathogenesis and progression of other diseases, such as diabetic neuropathy and Charcot–Marie–Tooth disease (CMT). In diabetic neuropathy, hyperglycemia, hypoxia, and oxidative stress collectively cause SC dysfunction, leading to myelin damage and deterioration of the peripheral nerve microenvironment 54. CMT is the most common inherited neuropathy. It is caused by demyelination and/or axonal damage due to a mutation or copy number abnormality at more than 70 gene loci that are vital for the development and activity of SCs and peripheral axons 55, 56. Therapeutic benefits may be obtained by transplantation of either allogenic dSCs or gene‐modified autologous dSCs. Meanwhile, dSCs may be also useful for analysis of pathogenesis and drug discovery of SC‐related diseases, because a large number of dSCs can be prepared from patients' fibroblasts.

## Author Contributions

Y.S.: Conception and design, Provision of study materials, Data analysis and interpretation, Manuscript writing; T.K.: Provision of study material, Data analysis and interpretation; K.T.: Provision of study materials; K.Y.: Data analysis and interpretation; T.N.: Administrative support; O.M.: Conception and design, Manuscript writing, Final approval of manuscript.

## Disclosure of Potential Conflicts of Interest

The authors indicate no potential conflicts of interest.

## Supporting information

Supporting Information

Supporting Information

Supporting Information

Supporting Information

Supporting Information
