# Supplementary material for: Direct Conversion of Human Fibroblasts into Schwann Cells that Facilitate Regeneration of Injured Peripheral Nerve In Vivo
Source: Stem Cells Transl Med. 2017 Jan 9;6(4):1207–16. doi: 10.1002/sctm.16-0122 (PMC5442846; doi:10.1002/sctm.16-0122)
Supplement: Supplementary file 1 — Supporting Information [file SCT3-6-1207-s001.pdf]

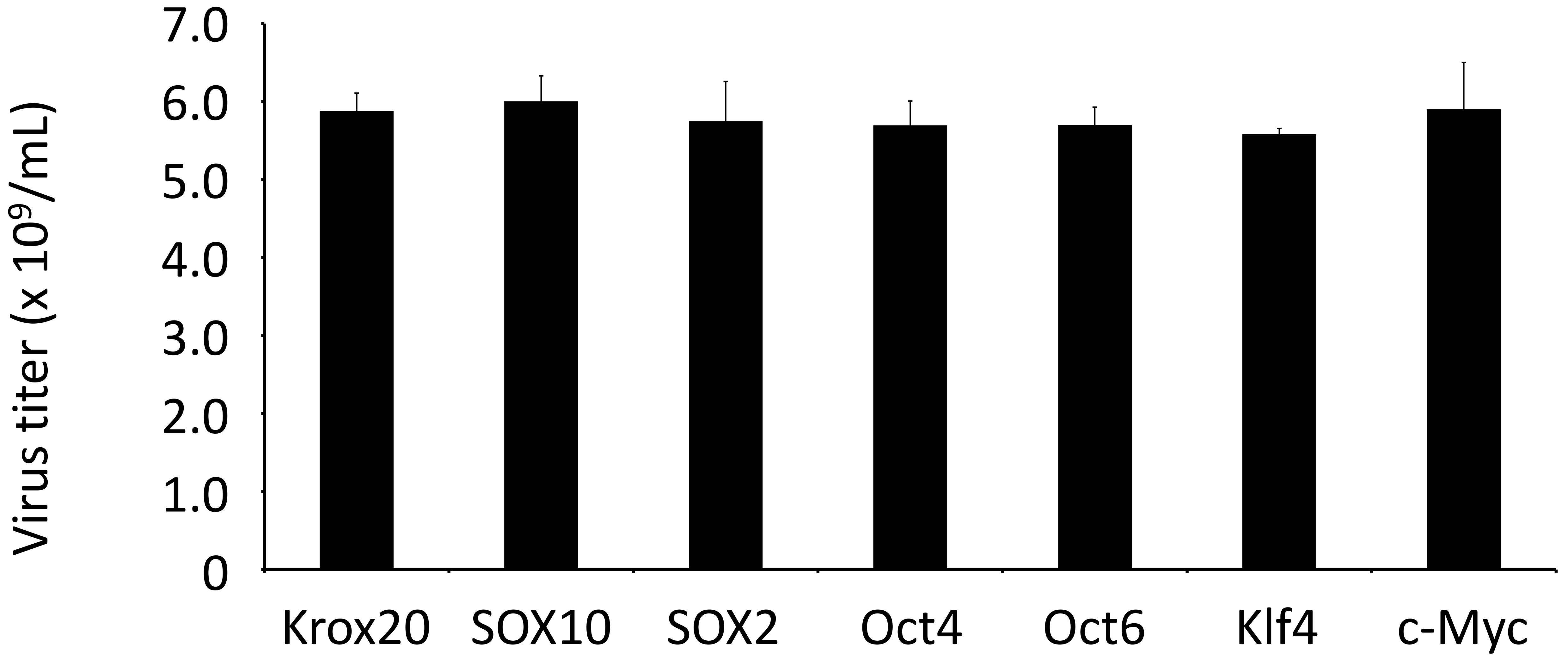

Supplementary Fig. S1

Each retroviral vector was prepared as described in the Materials and Methods. Virus titers were evaluated using the QuickTiter Retrovirus Quantitation Kit (Cell Biolabs) according to the manufacturer's instructions. Values are means ± SD. n=3 samples.

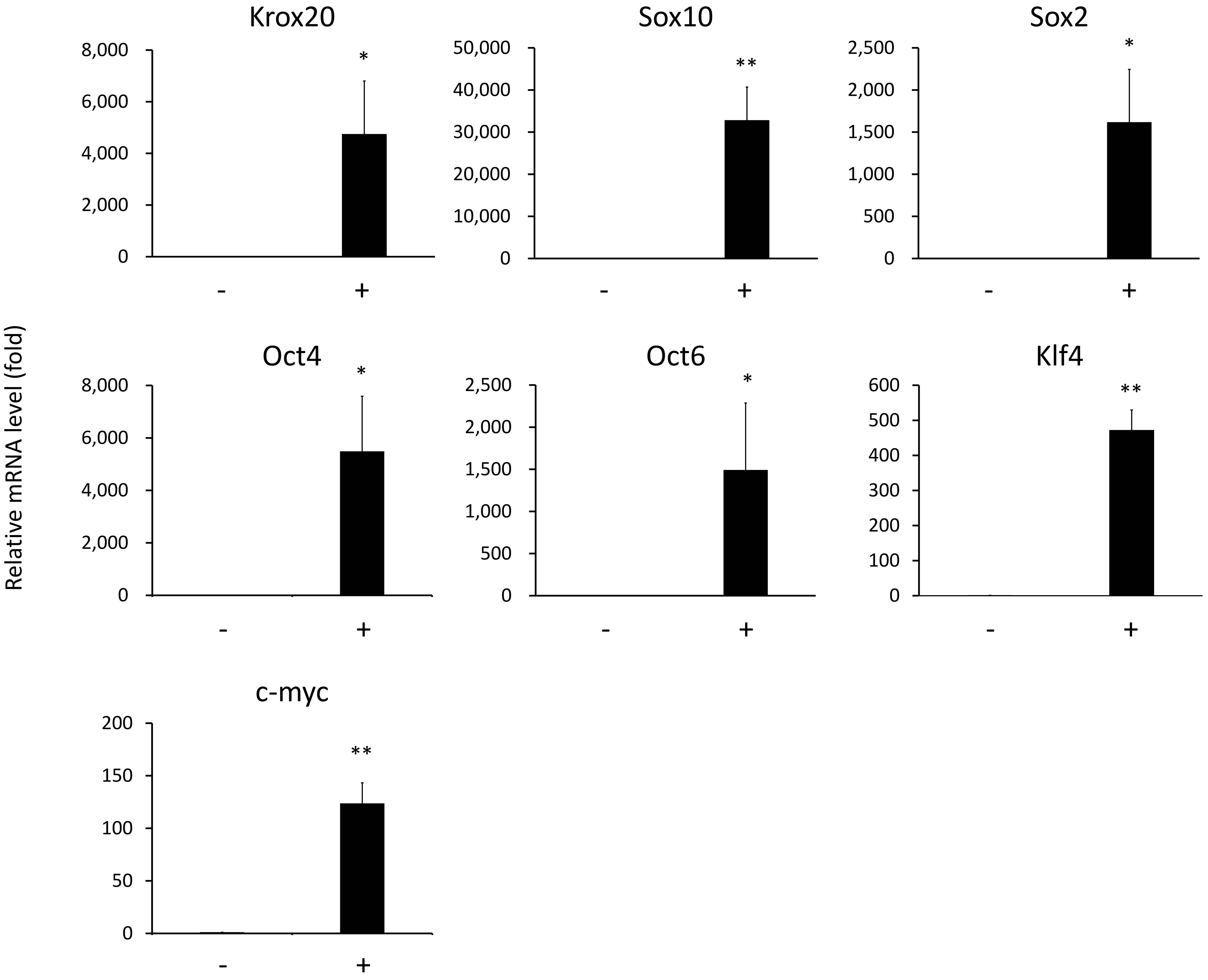

Supplementary Fig. S2

aHDFs were infected with a mixture of seven retroviral vectors (Krox20, Sox10, Sox2, Oct4, Oct6, , Klf4 and c-myc) as described in the Materials and Methods (+). Four days after the transduction, RNA was extracted from the cells, and mRNA level of each gene was evaluated by real time-RT-PCR. As control, mRNA level of each gene in uninfected fibroblasts (-) was also determined and set to 1.0. Values are means ± SD. n=3 cultures. *p<0.05 and **p<0.01 vs. Uninfected control. All the transgenes were strongly expressed in the cells co-transduced with the seven genes. As control, the other aliquot of aHDFs were not infected (-).

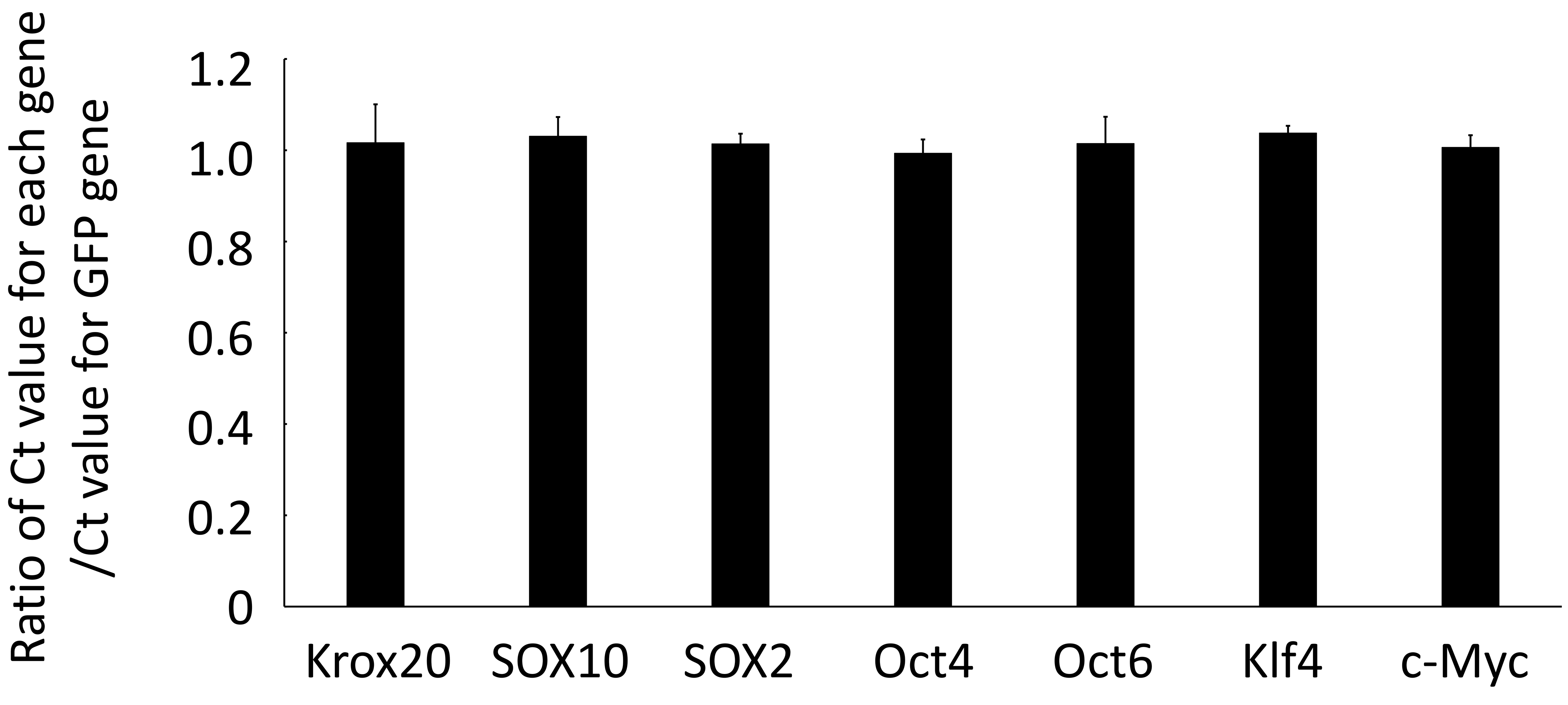

Supplementary Fig. S3

aHDFs were infected with a mixture of eight retroviral vectors (GFP, Sox10, Krox20, Sox2, Klf4, Oct4, Oct6, and c-myc) as described in the Materials and Methods. Four days after the transduction, RNA was extracted from the cells and subjected to real time-RT-PCR to determine Ct (threshold cycle) value for each gene. The ratio of (Ct value for each gene)/(Ct value for GFP gene) is shown. Values are means ± SD. n=3 cultures. All the transgenes were expressed in the co-transduced cells at similar levels.

Phase contrast	Green: GFP
Blue: DAPI	Merge

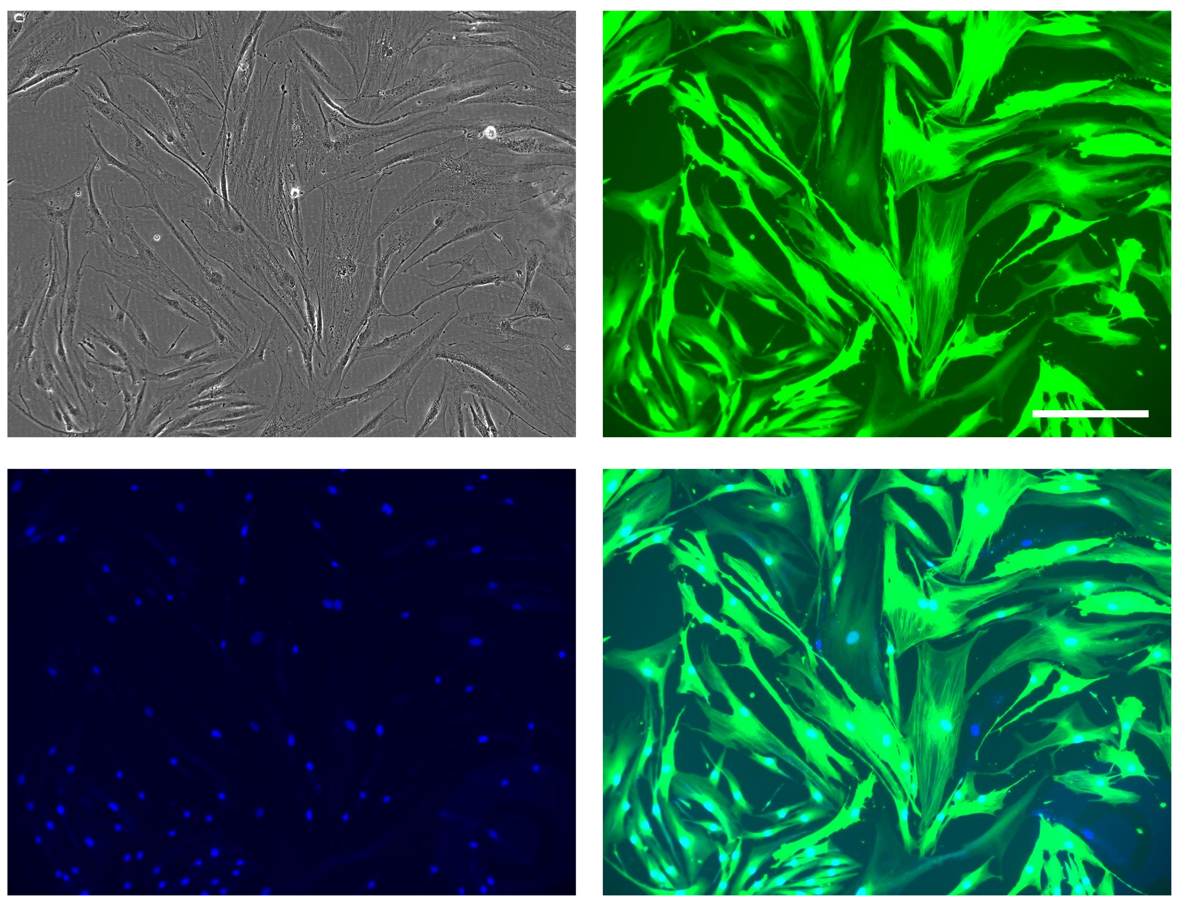

Supplementary Fig. S4

High transduction efficiency of pMX.GFP retroviral vector.

HDFs were infected with the pMX.GFP retroviral vector as described in the Materials and Methods. Four days later, cells were stained with the DAPI. Representative phase contrast (upper left), green and blue fluorescence (upper right and lower left, respectively) and merged (lower right) images are shown (magnification was ×100). Percentage of the (GFP-positive cells)/(total cells) was 95.1 ± 3.1% as calculated by the BZ-H3A software (Keyence). Scale bar=50 μm
