# Supplementary material for: Direct Conversion of Human Fibroblasts into Schwann Cells that Facilitate Regeneration of Injured Peripheral Nerve In Vivo
Source: Stem Cells Transl Med. 2017 Jan 9;6(4):1207–16. doi: 10.1002/sctm.16-0122 (PMC5442846; doi:10.1002/sctm.16-0122)
Supplement: Supplementary file 3 — Supporting Information [file SCT3-6-1207-s003.pdf]

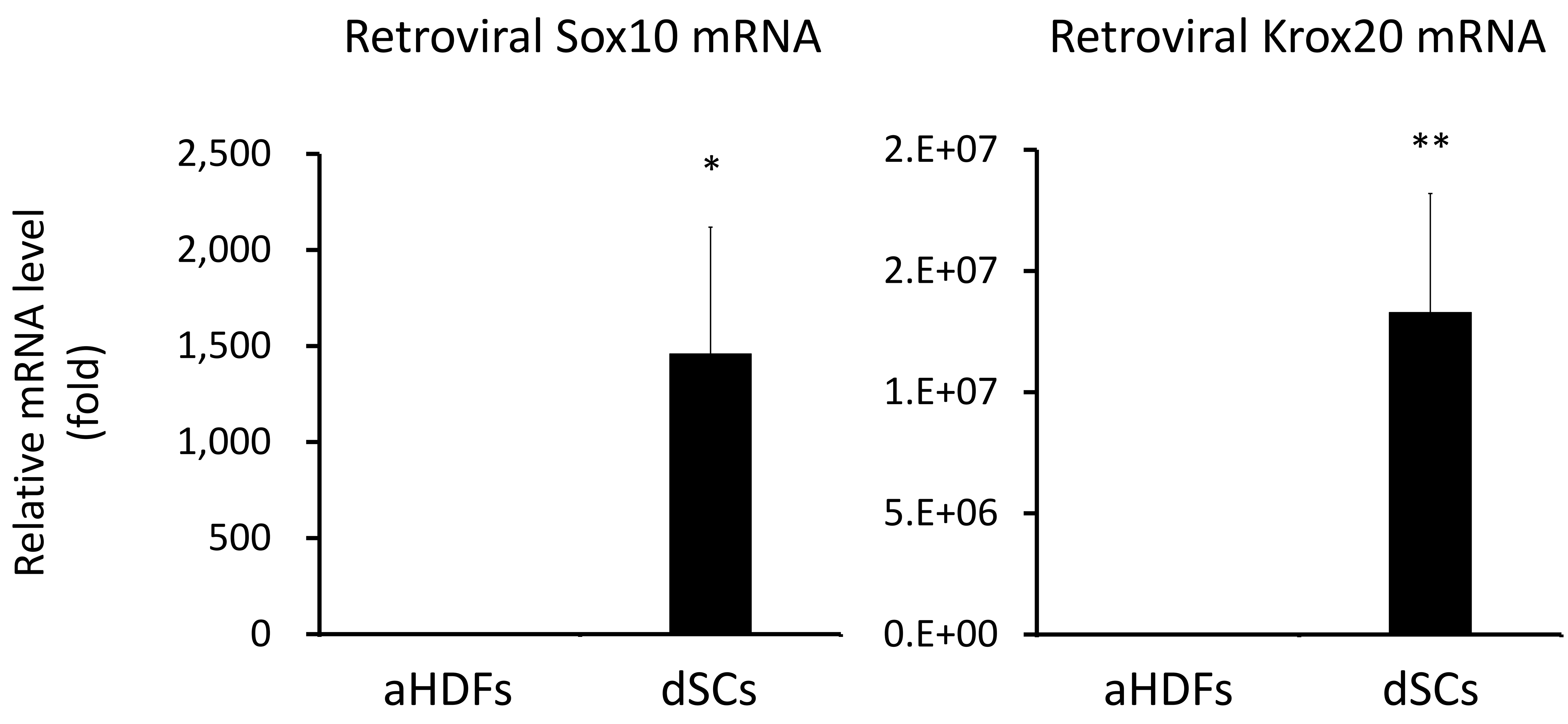

Supplementary Fig. S6

aHDFs were transduced with SOX10 and Krox20 genes as in Fig. 2. Fourteen days later, RNA was extracted from the cells (dSCs). As control, RNA was also extracted from untransduced aHDFs. Real time RT-PCR was performed to evaluate mRNA levels for the retroviral SOX10 and Krox20 transgenes. See Supplementary Table S1 for the real time RT-PCR primers/probes that specifically recognize transgenes. Values are means ± SD. n=3 cultures. *$p<0.05$, and **$p<0.01$ vs. aHDFs.

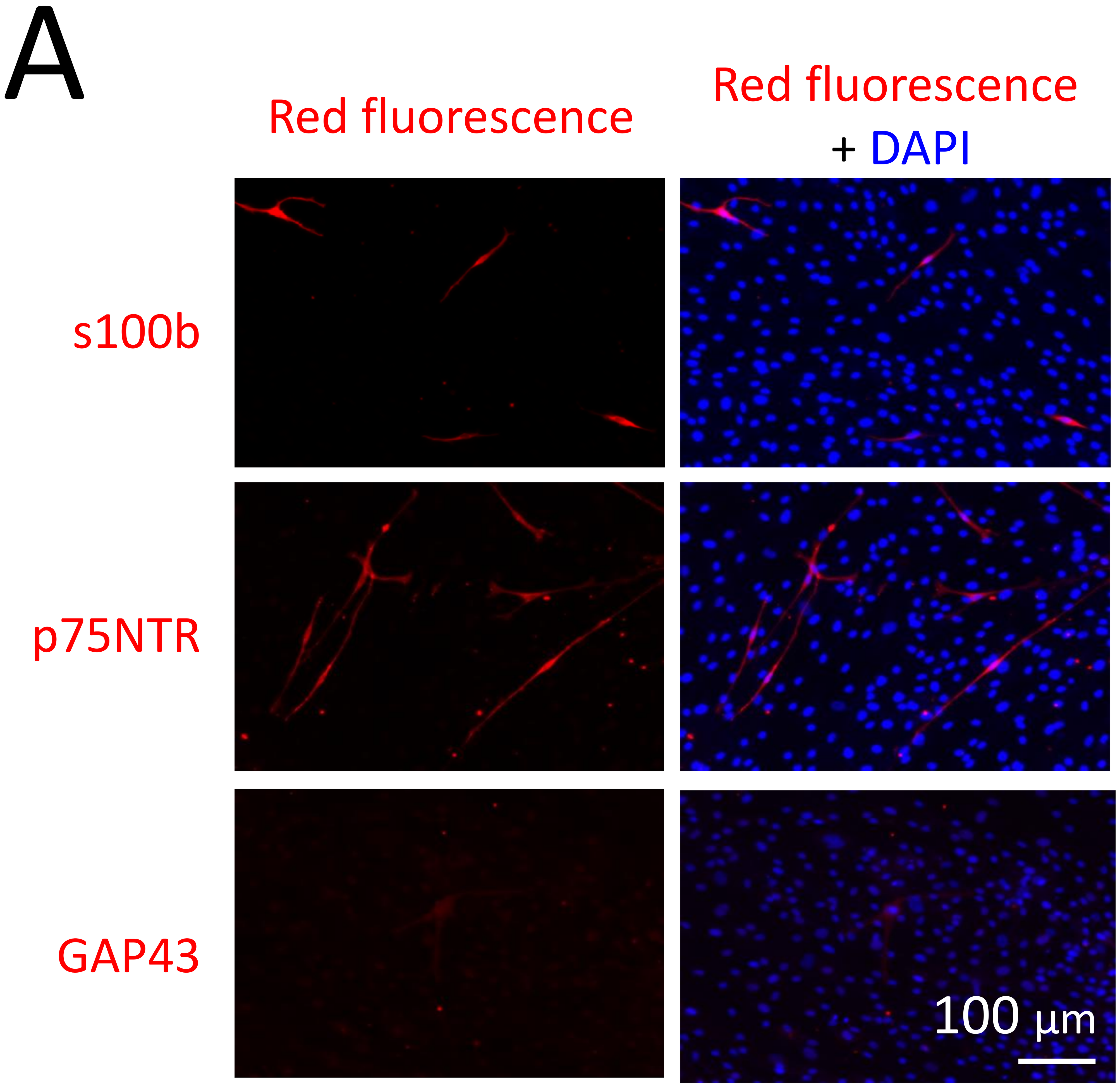

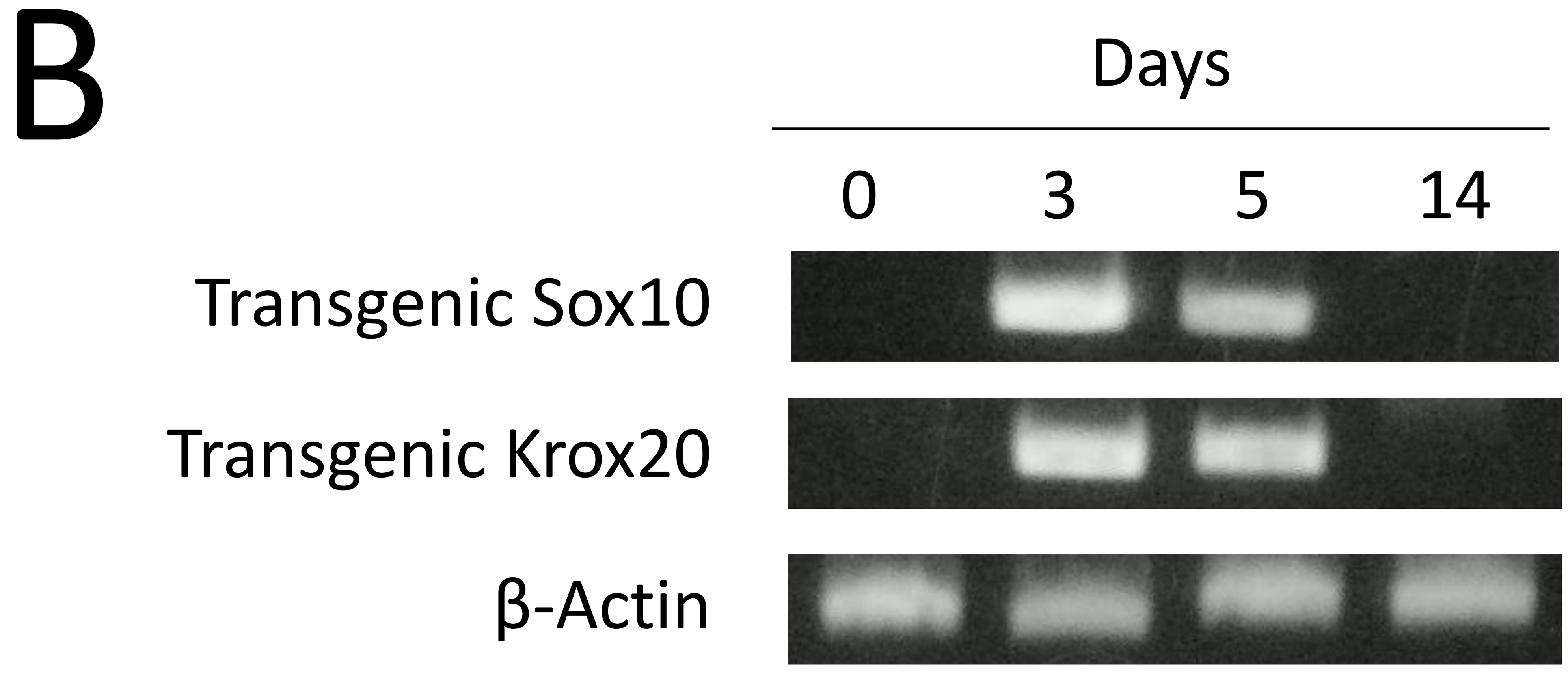

Supplementary Fig. S7

SC-like phenotypes were successfully induced by transient expression of SK genes. aHDFs were transfected with a plasmid vector containing the human SOX10 and Krox20 genes by electroporation, followed by culturing in SC medium. A, Fourteen days after transfection, cells were incubated with the indicated antibodies, secondary antibodies conjugated with Alexa Fluor 670, and DAPI. Representative red fluorescence (left) and merged (right) images are shown (magnification was × 100). B, Indicated days after transfection, RNA was extracted from the cells ,and the indicated DNA sequence was amplified by PCR using primers shown in Supplementary Table S2. dSCs maintained their phenotypes even after the plasmid DNA was lost from the cells.

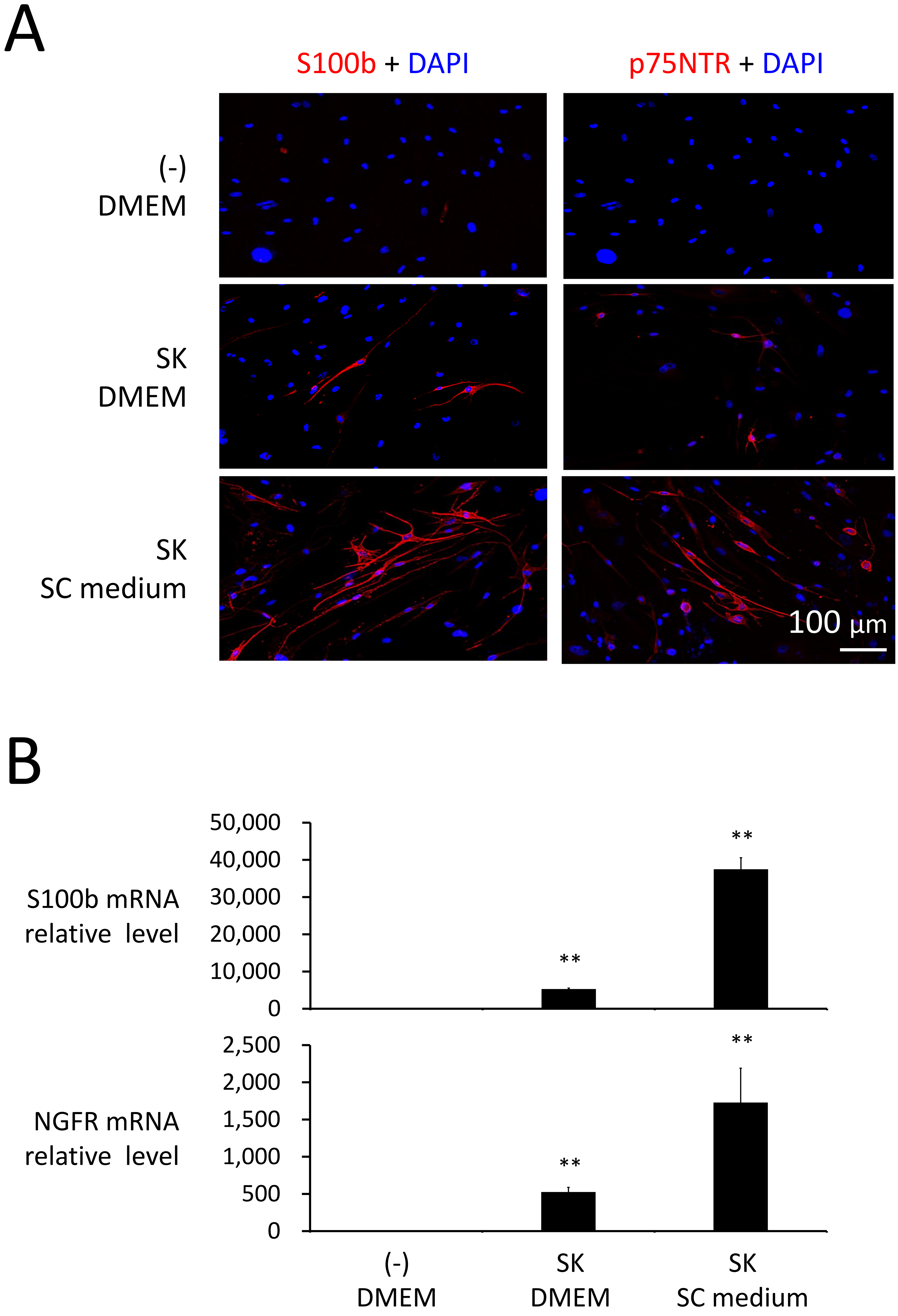

Supplementary Fig. S8
aHDFs were infected with SK retroviral vectors as in Fig. 2 (SK)(day 0), while other aliquots of cells were not infected as control (-). On the next day, culture medium was replaced by fresh DMEM supplemented with 10% FBS, 10 mM NEAA and antibiotics, or SC medium. The culture medium was changed every other day. (A) On day 16, cells were stained with either anti-S100b or anti-p75NTR antibodies and DAPI as in Fig. 1A. Red and blue merged florescence images are shown. (B) On day 14, RNA was extracted from the cells and S100b and NGFR mRNA was evaluated. mRNA level of uninfected cells was set to 1.0 for each gene. Values are means ± SD. n=3 cultures. **p<0.01 vs. Control.

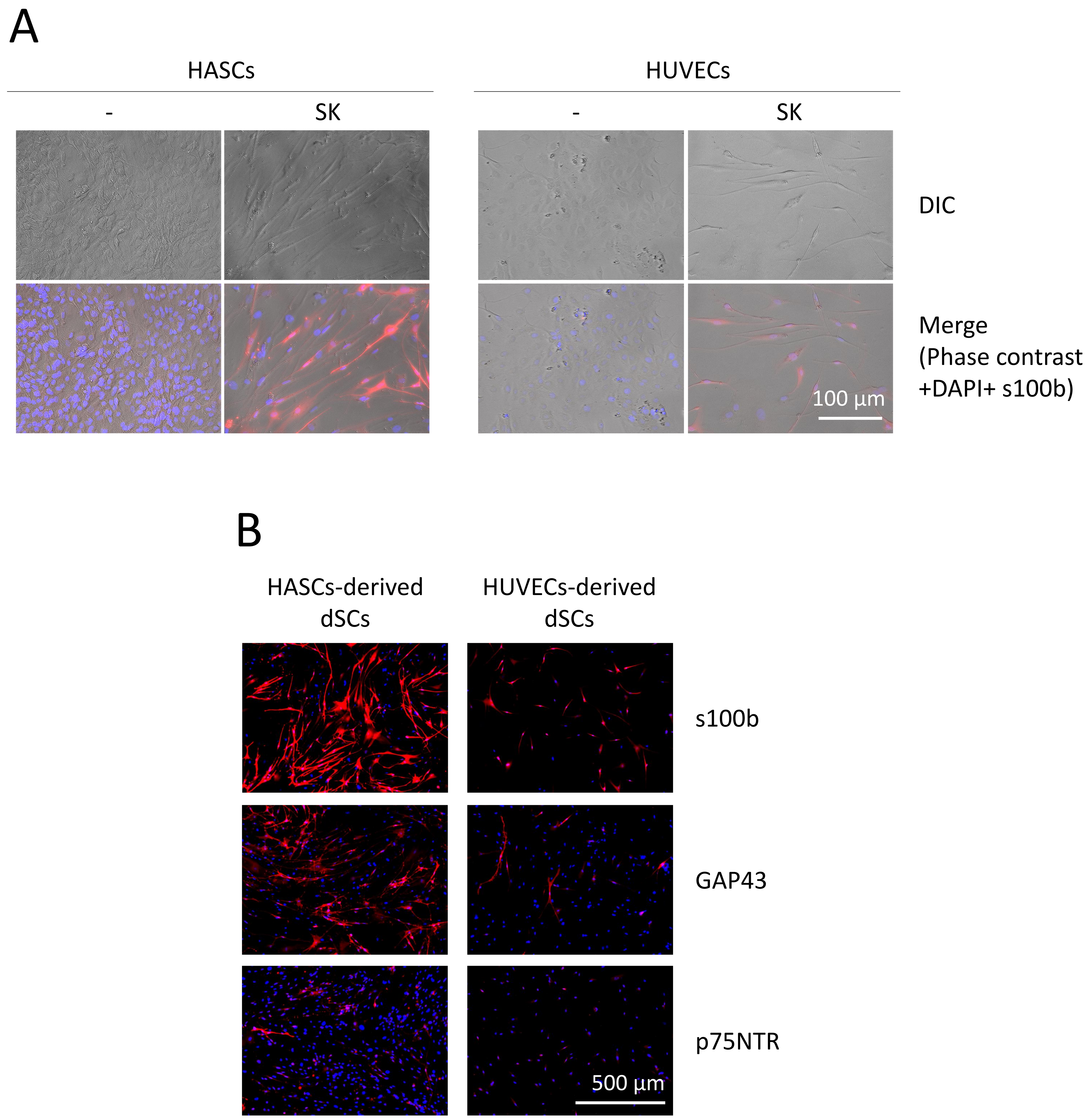

Supplementary Fig. S9

Human dSCs were induced from human adipose stromal cells (HASCs) and from human umbilical vein endothelial cells (HUVECs). (A) HASCs and HUVECs were infected with SK retroviral vectors or left un-infected (-), followed by culturing in SC medium as in Fig. 2. Ten days later, cells were stained with ant-s100b antibody and DAPI. Representative phase-contrast microscopic images (upper) and phase-contrast and fluorescence microscopic merged images (lower) are shown (magnification, x 200). (B) HASCs and HUVECs were infected with SK retroviral vectors and cultured as in (A). Representative fluorescence images of the cells stained with the indicated antibodies and DAPI are shown (magnification, x 100).

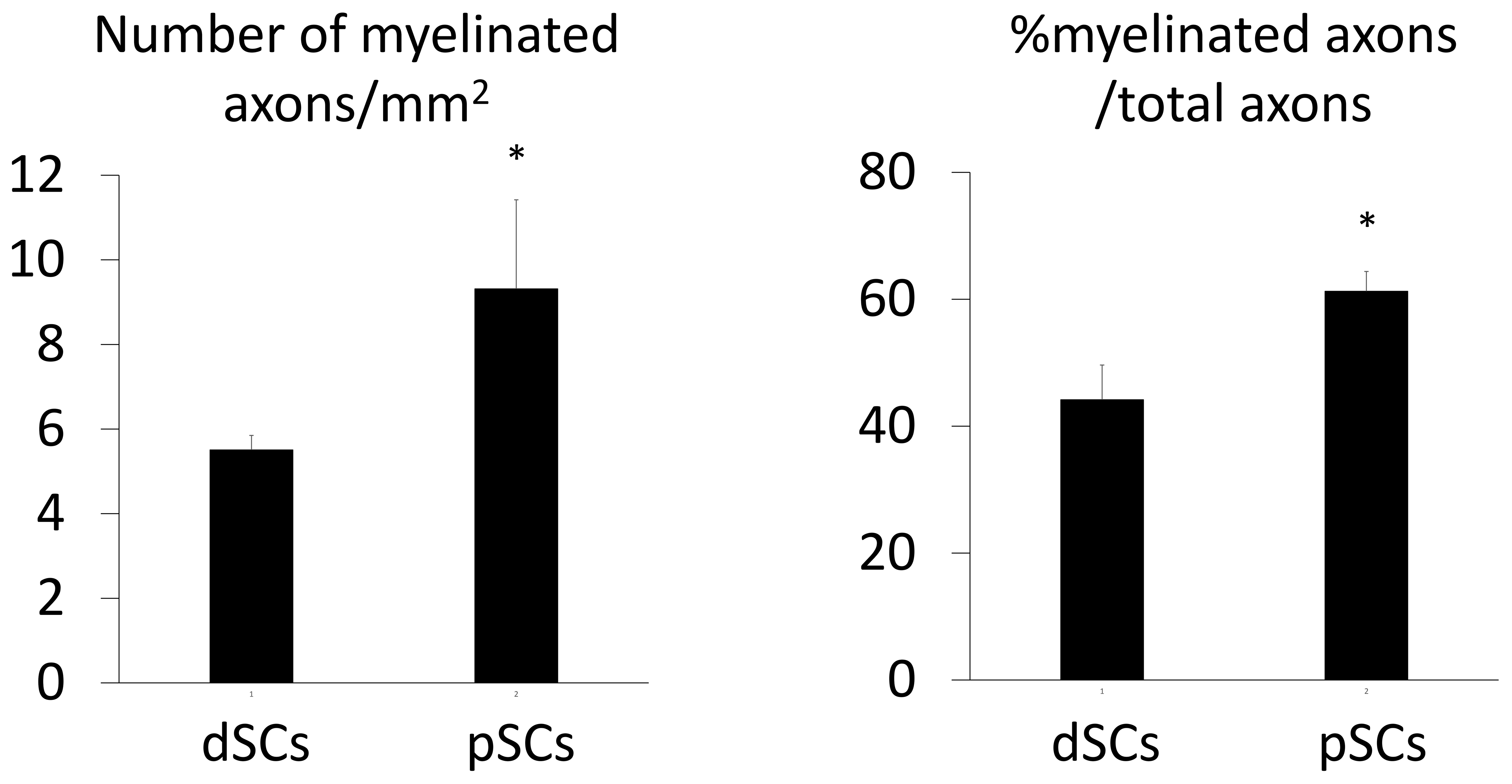

Supplementary Fig. S10

GFP-transduced dSCs were co-cultured with primary rat DRG for 14 days, and myelinated axons were visualized by staining MBP and Tuj1 as in Fig. 4B. Number of myelinated axon per mm^2 (left) and % (myelinated axons) /(total axons)(right) were calculated using Image J software. Values are means ± SD. n=3 cultures. *$p<0.05$ vs. Control.
