# Supplementary material for: Direct Conversion of Human Fibroblasts into Schwann Cells that Facilitate Regeneration of Injured Peripheral Nerve In Vivo
Source: Stem Cells Transl Med. 2017 Jan 9;6(4):1207–16. doi: 10.1002/sctm.16-0122 (PMC5442846; doi:10.1002/sctm.16-0122)
Supplement: Supplementary file 4 — Supporting Information [file SCT3-6-1207-s004.docx]

Supplementary Table S1　List of real-time RT-PCR primers and probes

| Target | Description/Sequences |
| --- | --- |
| hβ-actin | Applied Bioscience Hs00287164-m1 |
| hS100b | Applied Bioscience Hs00902901_a1 |
| hGAP43 | Applied Bioscience Hs00967138_m1 |
| hp75NTR | Applied Bioscience Hs00609977_m1 |
| hGFAP | Applied Bioscience Hs00909233_m1 |
| hSMBP | Applied Bioscience Hs00921945_m1 |
| hSOX2 | Applied Bioscience Hs01053049_s1 |
| hSOX10  (specific for endogenous SOX10) | Sense: acacggttttccacttcctaagga |
|  | Antisense: ctggaggagaggtcc |
|  | Probe: ttggactctttgcgaggacc |
| hKrox20  (specific for endogenous Krox20) | Sense: gagttgggtc tccaggttgtg |
|  | Antisense: cgtagacaaaatcc |
|  | Probe: tgtctgacaacatctacccggt |
| Retroviral SOX10 | Sense: ttaaggtcccagttggtggta |
|  | Antisense: atggcggaggagca |
|  | Probe: cctatcggaggtggagctga |
| Retroviral Krox20 | Sense: ttaaggtcccagttggtggta |
|  | Antisense: cgtagacaaaatcc |
|  | Probe: tgtctgacaacatctacccggt |
