# Supplementary material for: Direct Conversion of Human Fibroblasts into Schwann Cells that Facilitate Regeneration of Injured Peripheral Nerve In Vivo
Source: Stem Cells Transl Med. 2017 Jan 9;6(4):1207–16. doi: 10.1002/sctm.16-0122 (PMC5442846; doi:10.1002/sctm.16-0122)
Supplement: Supplementary file 5 — Supporting Information [file SCT3-6-1207-s005.docx]

Supplementary Table S2　List of PCR primers

| Target | | Sequences | |
| --- | --- | --- | --- |
| SOX10 plasmid | | Sense: cctttgtcccaaatctgtgc | |
|  |  | Antisense: acggggaacttgtcatcgt | |
| Krox20 plasmid | | Sense: ccccttctccctctccag | |
|  |  | Antisense: agggtcaatggagaacttgc | |
| β-actin | | Sense: gccatgtacgttgctatcca | |
|  |  | Antisense: gctggaagagtgcctcag | |
|  |  | |  |
