# Supplementary material for: Direct Conversion of Human Fibroblasts into Schwann Cells that Facilitate Regeneration of Injured Peripheral Nerve In Vivo
Source: Stem Cells Transl Med. 2017 Jan 9;6(4):1207–16. doi: 10.1002/sctm.16-0122 (PMC5442846; doi:10.1002/sctm.16-0122)
Supplement: Supplementary file 2 — Supporting Information [file SCT3-6-1207-s002.pdf]

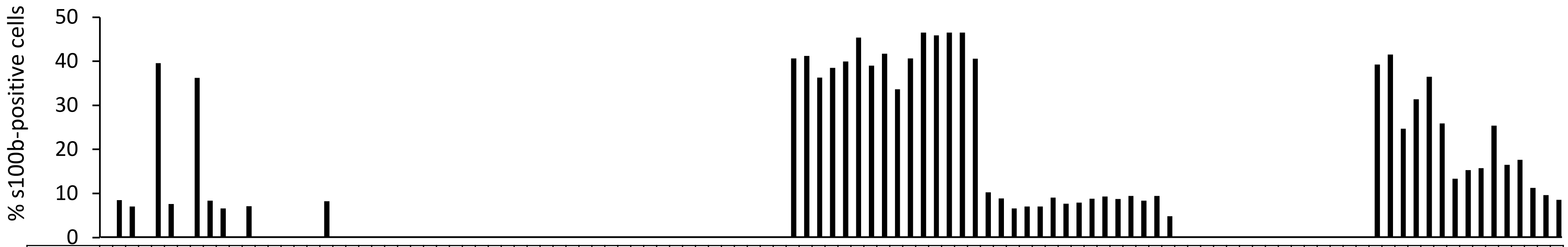

No.	0	1	2	3	4	5	6	7	8	9	10	11	12	13	14	15	16	17	18
SOX10		+			+	+		+	+	+	+	+	+	+	+	+	+	+	+
Krox20			+		+		+	+											
Oct6				+		+	+	+											
Sox2									+				+			+	+		+
c-Myc										+			+	+				+	+
KLF4											+			+	+	+			+
Oct4												+			+		+	+	

No.	19	20	21	22	23	24	25	26	27	28	29	30	31	32	33	34	35	36	37
SOX10	+	+	+	+															
Krox20					+	+	+	+	+	+	+	+	+	+	+	+	+	+	+
Oct6																			
Sox2	+	+		+	+				+			+	+		+	+	+		+
c-Myc	+		+	+		+			+	+				+	+	+		+	+
KLF4		+	+	+			+			+	+	+			+		+	+	+
Oct4	+	+	+	+				+			+		+	+		+	+	+	+

No.	38	39	40	41	42	43	44	45	46	47	48	49	50	51	52	53	54	55	56
SOX10																+	+	+	+
Krox20																+	+	+	+
Oct6	+	+	+	+	+	+	+	+	+	+	+	+	+	+	+				
Sox2	+				+			+	+		+	+	+		+	+			
c-Myc		+			+	+				+	+	+		+	+		+		
KLF4			+			+	+	+			+		+	+	+			+	
Oct4				+			+		+	+		+	+	+	+				+

No.	57	58	59	60	61	62	63	64	65	66	67	68	69	70	71	72	73	74	75
SOX10	+	+	+	+	+	+	+	+	+	+	+	+	+	+	+	+	+	+	+
Krox20	+	+	+	+	+	+	+	+	+	+	+								
Oct6												+	+	+	+	+	+	+	+
Sox2	+			+	+		+	+	+		+	+				+			+
c-Myc	+	+				+	+	+		+	+		+			+	+		
KLF4		+	+	+			+		+	+	+			+			+	+	+
Oct4			+		+	+		+	+	+	+				+			+	

No.	76	77	78	79	80	81	82	83	84	85	86	87	88	89	90	91	92	93	94
SOX10	+	+	+	+	+	+	+												
Krox20								+	+	+	+	+	+	+	+	+	+	+	+
Oct6	+	+	+	+	+	+	+	+	+	+	+	+	+	+	+	+	+	+	+
Sox2	+		+	+	+		+	+				+			+	+		+	+
c-Myc		+	+	+		+	+		+			+	+				+	+	+
KLF4			+		+	+	+			+			+	+	+			+	
Oct4	+	+		+	+	+	+				+			+		+	+		+

No.	95	96	97	98	99	100	101	102	103	104	105	106	107	108	109	110	111	112
SOX10				+	+	+	+	+	+	+	+	+	+	+	+	+	+	+
Krox20	+	+	+	+	+	+	+	+	+	+	+	+	+	+	+	+	+	+
Oct6	+	+	+	+	+	+	+	+	+	+	+	+	+	+	+	+	+	+
Sox2	+		+	+				+			+	+		+	+	+		+
c-Myc		+	+		+			+	+				+	+	+		+	+
KLF4	+	+	+			+			+	+	+			+		+	+	+
Oct4	+	+	+				+			+		+	+		+	+	+	+

Supplementary Fig. S5

Requirement of SOX10 and Krox20 for induction of s100b expression in human fibroblasts. aHDFs were transduced with mixtures of the indicated retroviral vectors (+). After culturing for 14 days, cells were stained with anti-s100b antibody and DAPI. %s100b-positive cells are plotted.
